# Mapping and characterizing areas with high levels of malaria in pregnancy in Brazil: A spatiotemporal analysis

**DOI:** 10.1016/j.lana.2022.100285

**Published:** 2022-05-27

**Authors:** Jamille Gregório Dombrowski, Laura Cordeiro Gomes, Camila Lorenz, Raquel Gardini Sanches Palasio, Paola Marchesini, Sabrina Epiphanio, Claudio Romero Farias Marinho

**Affiliations:** a Department of Parasitology, Institute of Biomedical Sciences, University of São Paulo, São Paulo, Brazil; b Department of Epidemiology, School of Public Health, University of São Paulo, São Paulo, Brazil; c Department of Transmissible Diseases Surveillance, Ministry of Health, Brasília, Brazil; d Department of Clinical and Toxicological Analyses, School of Pharmaceutical Sciences, University of São Paulo, São Paulo, Brazil

**Keywords:** Malaria, Pregnancy, Epidemiology, *P. vivax*, *P. falciparum*, Brazil

## Abstract

**Background:**

Malaria in pregnancy (MiP) is a public health problem in the Brazilian Amazon region that requires special attention due to associated serious adverse consequences, such as low birth weight, increased prematurity and spontaneous abortion rates. In Brazil, there have been no comprehensive epidemiological studies of MiP. In this study, we aimed to explore the spatial and spatiotemporal patterns of MiP in Brazil and epidemiologically characterize this population of pregnant women over a period of 15 years.

**Methods:**

We performed a national-scale ecological analysis using a Bayesian space-time hierarchical model to estimate the incidence rates of MiP between 1 January 2004 and 31 December 2018. We mapped the high-incidence clusters among pregnant women aged 10-49 years old using a Poisson model, and we characterized the population based on data from the Epidemiological Surveillance Information System for Malaria (SIVEP-Malaria).

**Findings:**

We consolidated the data of 61,833 women with MiP in Brazil. Our results showed a reduction of 50·1% (95% CI: 47·3 to 52·9) in the number of malaria cases over the period analysed, with *Plasmodium vivax* malaria having the highest incidence. MiP was widely distributed throughout the Amazon region, and spatial and spatiotemporal analyses revealed statistically significant clusters in some municipalities of Amazonas, Acre, Rondônia and Pará. In addition, we observed that younger pregnant women had a higher risk of infection, and the administration of appropriate treatment requires more attention.

**Interpretation:**

This nationwide study provides robust evidence that, despite the reduction in the number of MiP cases in the country, it remains a serious public health problem, especially for young pregnant women. Our analyses highlight focus areas for strengthening interventions to control and eliminate MiP.

**Funding:**

FAPESP and CNPq - Brazil.

**Research in context**

**Evidence before this study**

Malaria in pregnancy (MiP) imposes a significant burden on maternal and foetal health, as well as on the health systems of impoverished areas. *Plasmodium* infection during pregnancy leads to underweight babies, preterm deliveries, abortions, stillbirths, and even maternal mortality. We searched PubMed for articles published through January 15, 2021, without date or language restrictions that addressed or reported epidemiological data on MiP in Brazil, using the search terms “epidemiology” [All Fields] OR control” [All fields] OR “Brazil” [All fields] AND “malaria” [All Fields] OR “malaria in pregnancy” [All fields]. Only one study, published in 2014, on the epidemiology of MiP in Brazil was found, and it presented only an overview, without any detailed information. Although there have been several studies on MiP in Brazil, all of them have been performed in specific areas or cities and thus did not provide a nationwide overview of the disease. Importantly, none of these studies provided detailed epidemiological information on MiP in Brazil, nor did they account for the subnational variation in the distribution of infection in a fifteen-year historical series (2004-2018).

**Added value of this study**

We compiled a georeferenced *Plasmodium* infection incidence dataset from Malaria Epidemiological Surveillance Information System data (SIVEP-Malaria) from the Ministry of Health of Brazil. This work provides a complete overview of the epidemiology of MiP throughout Brazil, showing the evolution of the disease over fifteen years, from 2004-2018. In addition, the data were analysed to characterize the disease according to parasite species and location of infection. This is the first work to provide a complete description of the burden of gestational malaria in Brazil.

**Implications of all the available evidence**

Although the overall MiP prevalence substantially declined from 2004 to 2018, not all cities followed this trend. This time series analysis quantifies the evolution of the disease since 2004. National malaria control programmes can use these geospatial data to identify areas that may require additional surveillance or interventions, acting on strategies to protect pregnant women and their babies from the devastating consequences of malaria infection.

Alt-text: Unlabelled box

## Introduction

Malaria in pregnancy (MiP) is a serious public health problem in several countries worldwide. Endemic to sub-Saharan Africa, Latin America, the Mediterranean, and Southeast Asia, *Plasmodium* spp. infection during pregnancy has serious consequences, such as low birth weight, prematurity, spontaneous abortion, stillbirth, maternal anaemia, and even maternal and perinatal mortality.^1^, ^2^, ^3^, ^4^ According to the World Health Organization Report, approximately 12 million pregnant women on the African continent, a region of high transmission of *Plasmodium falciparum*, developed malaria in 2019.^2^ However, epidemiological studies estimating the population of pregnant women affected by malaria have been scarce in regions with low transmission rates, such as Latin America, where *P. vivax* and *P. falciparum* coexist.

Only three countries in Latin America account for almost 90% of all reported malaria cases in the region: Venezuela (55%), Brazil (22%), and Colombia (11%).^2^ In Brazil, approximately 200 thousand cases have been registered in the country in recent years.^2^^,^^5^ Approximately 99% of these infections were concentrated in the Legal Amazon region.^5^, ^6^, ^7^ *P. vivax* infections are prevalent in the country, accounting for approximately 80% of reported cases, followed by *P. falciparum* infections.^7^ One-off studies have reported an incidence of gestational malaria of less than 10%.^7^, ^8^, ^9^ However, these studies were performed in single cities or in small regions, and it was not possible to infer the incidence of MiP throughout the country.

In Brazil, malaria cases are reported using the Malaria Epidemiological Surveillance Information System (SIVEP-Malaria) in malaria-endemic areas – the Amazonian region – and the Notifiable Diseases Information System (SINAN) in nonendemic areas. The SIVEP-Malaria was implemented in 2003 by the Health Surveillance Secretariat (SVS), and its main objective is to improve the flow and quality of information throughout the country.^8^ It is through these information systems - SIVEP-Malaria and SINAN - that all diagnosed malaria cases are reported, and the patient receives treatment free of charge. For pregnant women, the Brazilian Ministry of Health implemented a measure in 2014 in which a diagnostic test for malaria (thick drop, gold standard) was performed at all antenatal visits and at the time of delivery for women residing in the Amazon region.^8^ Therefore, early diagnosis and timely treatment are possible, and the devastating adverse effects of the disease can be prevented.

In recent decades, the country has recorded an important reduction in the annual number of malaria cases, mainly starting after 2007. According to the Brazilian Ministry of Health, the number of cases recorded in 2014 was the lowest in the previous 35 years, and between 150 thousand and 200 thousand notifiable cases have been reported in the last 3 years.^6^ However, despite advancements, it is estimated that more than 9 million women residing in the Amazon region are exposed to both *P. vivax* and *P. falciparum* and therefore have an increased risk of acquiring malaria and suffering from its effects. Thus, malaria continues to be an important public health issue in Brazil, with a significant impact on the economic development of this region. Therefore, the aim of this study was to describe the epidemiological characteristics of MiP in Brazil in a 15-year historical series (2004-2018) and to identify significant spatial and spatiotemporal clusters of malaria based on secondary information extracted from Brazilian information systems.

## Methods

### Study design and data source

We performed a national-scale ecological analysis using a Bayesian space-time hierarchical model to estimate the incidence rates of MiP, and we mapped the clusters with the highest incidence rates of the disease among pregnant women aged 10-49 years old between 1 January 2004 and 31 December 2018. Furthermore, a descriptive epidemiological study was conducted using secondary information extracted from Brazilian information systems. A detailed description of the study area can be found in the *Appendix,* p 2. The analysis included all reported cases of MiP in the municipalities of residence during the study period. Microscopy, using thick blood smears, is considered the gold standard for the diagnosis of malaria in Brazil. Since approximately 90% of malaria cases during pregnancy occurred in the place of residence (same infection site) (*Appendix*, p 3), this variable was used for standardization with the other information systems used in the study.

Ethical approval was not required for this research due to the public and anonymous nature of the data. This analysis adhered to the Guidelines for Accurate and Transparent Health Estimates Reporting - GATHER (*Appendix*, p 4).

Data were extracted mainly from the Epidemiological Surveillance Information System for Malaria (SIVEP-Malaria), an online information system of the SVS in Brazil that stores records of all malaria notification forms submitted since 2003. Other information systems were also used, such as the Mortality Information System (SIM), the Information System of Live Births (SINASC) and data from the Brazilian Institute of Geography and Statistics (IBGE), to calculate estimations of the number of pregnant and nonpregnant women in the analysed period (details in *Appendix*, p 5). Notifications of malaria cases outside the Amazon region were obtained from the SINAN from January 2007 to December 2018.

SIVEP-Malaria datasets from 2004 to 2014 were obtained directly from the SVS of the Ministry of Health after signing a document that guaranteed the confidentiality of the information by the researchers. From 2015 to 2018, due to a new information access law (Law no. 12.527, 18 November 2011), the datasets were obtained by request from the following website: https://esic.cgu.gov.br/sistema/site/index.aspx. The year 2004 was chosen as the initial year because it was the year preceding a major epidemic in the country and one year after the implementation of the SIVEP-Malaria throughout the Amazon region, where approximately 99% of malaria cases are concentrated in Brazil. Thus, 2003 was excluded due to the instability and lack of coverage related to the rollout of the system. Importantly, the notification form was updated in 2012, with the insertion and reformulation of variables (e.g., insertion of the variable “pregnancy trimester”). Therefore, the datasets were standardized, and only common variables were used for all years (*Appendix*, p 5).

The variables used in this study were based on the items on the SVS standardized malaria notification form. Initially, the dataset was cleaned, and the following variables were excluded: 1) male or undetermined sex; 2) age younger than 10 years old and older than 49 years old [analysis performed in only women of childbearing age]; 3) cure verification by thick smear (removed to avoid multiple counts of single cases); 4) nonpregnant status; 5) residence outside Brazil; and 6) infection contracted outside Brazil. In addition, variables with any inconsistencies, such as typos or missing information, were excluded. Details of the database clean-up process are provided in the *Appendix*, pp 6-7.

### Statistical analysis

Geospatial data were obtained to assess the distribution of malaria cases. To curb variance instability and reduce the occurrence of spurious values in areas with small populations, we used the spatial empirical Bayesian (SEB) smoothing method to smooth and map the crude incidence rates.^10^ The SEB smoothing method defines a neighbourhood for each spatial area; spatial smoothing is conducted based on the neighbourhood scale, the neighbourhood incidence, and the mean and variance of the neighbourhood area. As a result, the incidence of the disease can be recalculated by the SEB method.^11^ We achieved this goal by first creating a contiguity-based second-order rook spatial weights file (lower orders inclusive) covering all municipalities in the study area with the weights manager.^12^

Spatial and spatiotemporal clustering analyses of malaria incidence were performed. Retrospective spatial and space-time scan statistics were calculated to detect areas and periods with higher-than-expected malaria incidence rates (hotspots/clusters). To scan for high incidence rates, a Poisson probability model was used^13^ under the following conditions: maximum size of each cluster equal to 50% of the population and cluster with circular format. SaTScan usually detects the most likely clusters and secondary clusters of similar significance. To avoid possible geographical overlap among clusters, the criteria for reporting hierarchical clusters were defined as “no geographical overlap” (for spatiotemporal analysis) and “Gini indices” (for only spatial analysis). We delimited the maximum cluster size to 300 km to obtain only the most likely clusters among the smaller clusters, instead of a single large cluster containing all, considering that the study area has a large territorial extension, an area of 5,015,067.75 km², corresponding to approximately 58.9% of Brazilian territory.^14^ To increase the statistical power of our analysis and ensure that the p values produced had a finite number of decimals, we set the number of Monte Carlo replications to 999.^13^ Finally, clusters with a p value <0·05 were considered statistically significant.

Relative risks (RRs) and 95% confidence intervals (CIs) were calculated to estimate the association of each age group with malaria cases. The total number of pregnant women by municipality of maternal residence and year was used in the denominator. Descriptive statistics were performed using Stata (version 14.2) or GraphPad Prism (version 6.0) software. For spatial and spatiotemporal analyses, GeoDa (version 1.20) and SaTScan (version 9.6) software were used.

### Role of the funding source

The funder of the study had no role in the study design, data collection, data analysis, data interpretation, or writing of the report. The corresponding author had full access to all the data in the study and had final responsibility for the decision to submit for publication.

## Results

### Baseline characteristics

Between 2004 and 2018, approximately 61,833 cases of MiP were reported across the Amazon region. Table 1 summarizes all sociodemographic and infection information of pregnant women from the notification forms. In general, pregnant women were young, with a median age of 22 years old; most had more than five years of education, and 38% said they were housewives (occupation in the last 15 days). Regarding infection information, we observed that almost 75% of the notifications were collected passively; that is, the woman went to a health unit to undergo a diagnostic exam.

**Table 1:** Sociodemographic and infection characteristics of non-pregnant women of childbearing age and the population of pregnant women, 2004-2018.

	Population of infected nonpregnant womenN=1,007,094	Population of infected pregnant womenN=61,833
**Age, years**	23 (15-33)	22 (18-27)
**Education level, n (%)**		
No education	87,477 (9·8)	5,831 (10·4)
Primary	338,102 (37·8)	19,797 (35·2)
≥ Secondary	469,328 (52·4)	30,598 (54·4)
Occupation*		
Agriculture	169,208 (19·9)	11,286 (20·2)
Housewife	228,071 (26·8)	21,247 (38·0)
Others/Ignored	452,611 (53·3)	23,391 (41·8)
**Detection type, n (%)**		
Active	271,052 (26·9)	15,670 (25·3)
Passive	736,042 (73·1)	46,163 (74·7)
**Species of parasite, n (%)**		
*P. vivax*	815,156 (80·9)	47,867 (77·4)
*P. falciparum*	178,662 (17·7)	12,990 (21·0)
Others	13,276 (1·3)	976 (1·6)
**Presence of symptoms, n (%)**	950,493 (94·4)	58,244 (94·2)
**Correct treatment**		
*P. vivax*	754,171 (74·9)	7,778 (12·6)
*P. falciparum*	108,372 (10·8)	3,486 (5·6)
Other scheme used (by medical prescription)	59,897 (6·0)	10,811 (17·5)
No information	59,114 (5·9)	4,963 (8·0)

Abbreviations: N, total number of individuals; *P, Plasmodium*. The results are presented as the median and interquartile range or total number of events (n) and percentage (%). *Occupation in the last 15 days declared at the time of notification. There were 112,187 missing values for education level and 157,204 missing values for occupation in the nonpregnant women group; and there were 5,607 missing values in education level and 5,909 missing values in occupation in the infected pregnant women group.

Regarding species, *P. vivax* was the most prevalent species (77·4%), followed by *P. falciparum* (21·0%). The presence of symptoms was quite common, with 94·2% reporting feeling some symptoms; however, only 18·2% of the pregnant women had the appropriate treatment notification (Table 1). In addition, when comparing the data of pregnant women with the total data of women of childbearing age, there was great similarity between the groups in terms of sociodemographic and infection characteristics, except for the notification of correct treatment, which increased to 85·7% (Table 1). Pregnant women represented approximately 5·8% of the total cases and had a higher frequency of *P. falciparum* malaria than nonpregnant women (*Appendix*, p 8).

### Evolution of MiP

**Figure 1:**
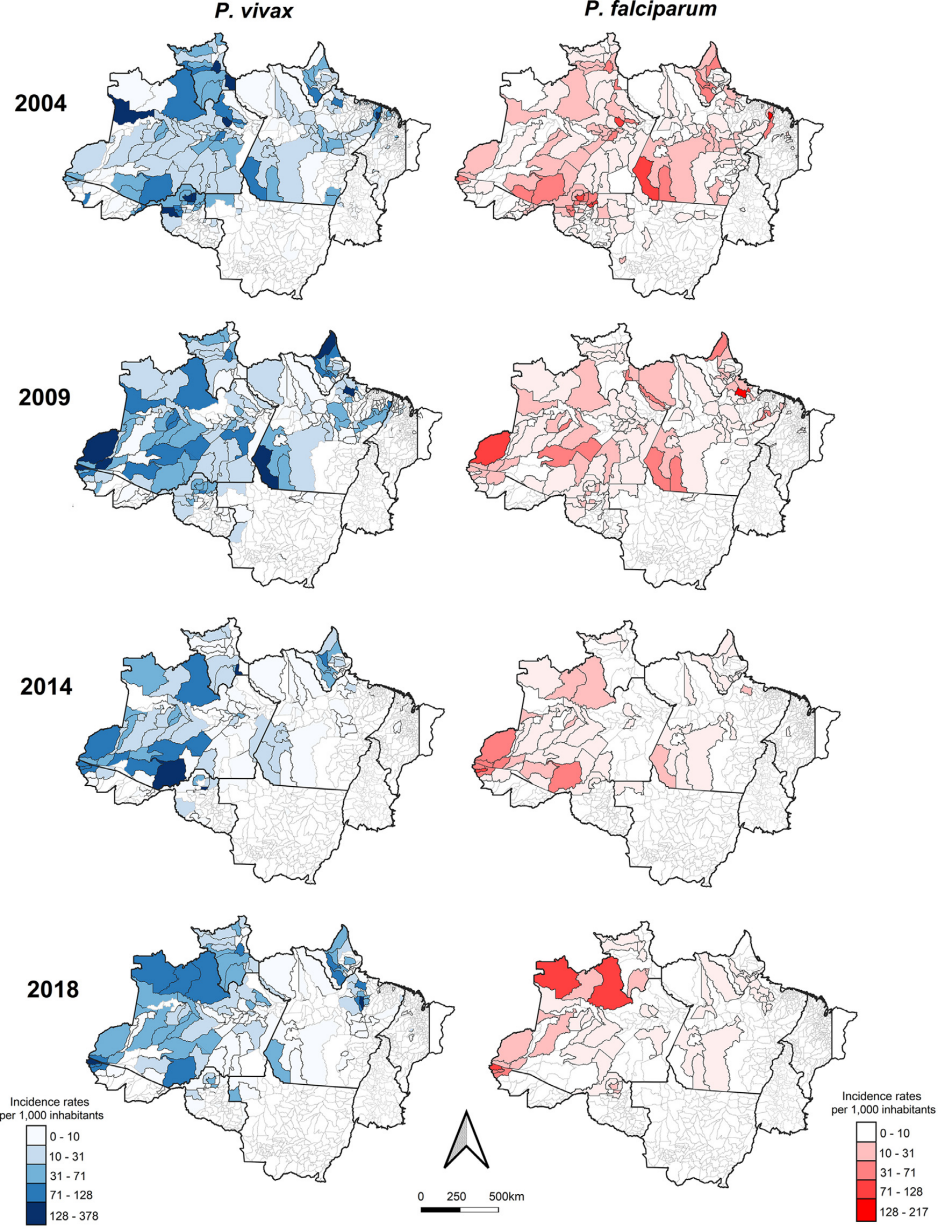
Spatial empirical smoothed Bayesian analysis of gestational malaria incidence rates per 1000 inhabitants by municipality and *Plasmodium* species. Brazilian Legal Amazon, Brazil.

Figure 1 shows the spatiotemporal distribution of the incidence of gestational malaria in the Amazonian municipalities. Although the entire region is considered endemic, the number of municipalities that reported both *P. vivax* and *P. falciparum* malaria cases decreased over the years. Regarding malaria caused by *P. vivax*, in 2004, 14 municipalities showed an annual incidence greater than 100 cases/1000 pregnant women. The peak incidence in the region occurred in 2006, when 27 municipalities had an incidence rate greater than 100, with the highest incidence rate being 496·9 cases/one thousand pregnant women in Mâncio Lima (Acre State). In the following years, there was a decrease in the total incidence rate, with only 4 municipalities reporting incidence rates greater than 100 in 2018. *P. falciparum* malaria also exhibited a trend of decreasing incidence over the years. The peak was also in 2006, with 6 municipalities reporting incidence rates greater than 100 cases/1000 pregnant women, and the highest incidence was 431·7 in the same municipality of Mâncio Lima (Acre State). From 2013, no municipalities reported incidence rates greater than 100 for *P. falciparum* malaria. Although there were downwards trends in the incidence rates, there were still some municipalities with a high risk of transmission in 2018, and cases of malaria caused by both species persisted mainly in the northern region of the State of Amazonas (Figure 1). The maps with the distribution of cases by species for all years can be found in the *Appendix*, pp 9-10.

### Decreasing number of MiP cases over the years

**Figure 2:**
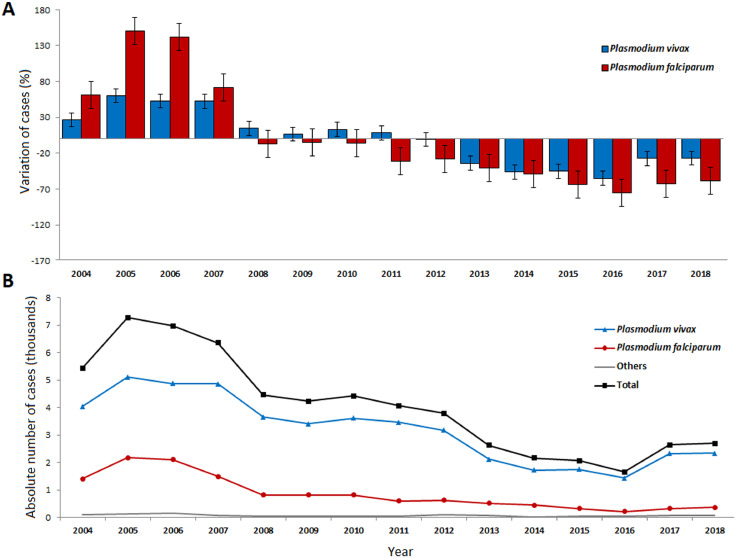
Distribution of notified malaria cases among pregnant women aged 10–49 years old in the Brazilian Legal Amazon, 2004–2018. (A) Percentage of variation in cases of malaria in pregnancy by year and species with the highest incidence rates. (B) Annual distribution of malaria cases by parasite species.

The historical series showed a substantial decrease in the number of cases, especially between 2008 and 2016 (Figure 2A). However, from 2016 onwards, there was a reversal of this trend, with increases in the numbers of cases in 2017 and 2018 (Figure 2). Despite the three epidemic peaks in the period analysed (2005, 2010, and 2017), the number of cases in Brazil decreased from 5520 in 2004 to approximately 2756 in 2018, representing a reduction of 50·1% (95% CI 47·3 to 52·9) (Figure 2B).

Figure 2 shows the dynamic behaviour of malaria over the years, but monthly assessments are also important. Since it is known that the seasonality of malaria is different between states, although they all belong to the northern region of Brazil, we performed monthly analyses for the last 3 years (2016-18) for the 6 states that reported the highest numbers of malaria cases (***Appendix*, p 11**). We observed that the monthly distribution was not homogeneous and that it also differed between states. When compared with malaria in the population of nonpregnant women, we observed that, in most states, malaria in pregnant women had a different profile throughout the year, suggesting that malaria in this population does not follow the endemic profile of that in the general population (*Appendix*, p 11).

### Endemicity of states in the Brazilian Legal Amazon

Table 2 shows the number of malaria cases in pregnant women by state of residence during the period analysed and species of parasite. All states except Acre had a *P. falciparum* infection percentage of approximately 20%. Acre State had the highest incidence of *P. falciparum* infection, representing approximately 30% of the total recorded cases of gestational malaria.

**Table 2:** Distribution of malaria cases in pregnant women in the Brazilian Legal Amazon according to state of residence.

	*P. vivax*		*P. falciparum*		Mixed and others		Total	
	N	%	N	%	N	%	N	%
**Acre**	5226	68·9	2273	30·0	88	1·2	7587	12·3
**Amapá**	2901	80·0	668	18·4	74	2·0	3643	5·9
**Amazonas**	19846	78·8	5030	20·0	294	1·2	25170	40·7
**Maranhão**	616	80·9	144	18·9	2	0·3	762	1·2
**Mato Grosso**	333	75·0	105	23·6	6	1·4	444	0·7
**Pará**	12168	77·0	3233	20·5	386	2·4	15787	25·5
**Rondônia**	4924	79·8	1164	18·9	88	1·4	6176	10·0
**Roraima**	1842	81·7	370	16·4	38	1·7	2250	3·6
**Tocantins**	11	78·6	3	21·4	-	0	14	0
**Total**	47867	77·4	12990	21·0	976	1·6	61833	100·0

Abbreviations: N, total number of individuals; P, *Plasmodium*. The results are presented as the total number of events and percentage (%).

### Spatial and spatiotemporal clusters of MiP

**Figure 3:**
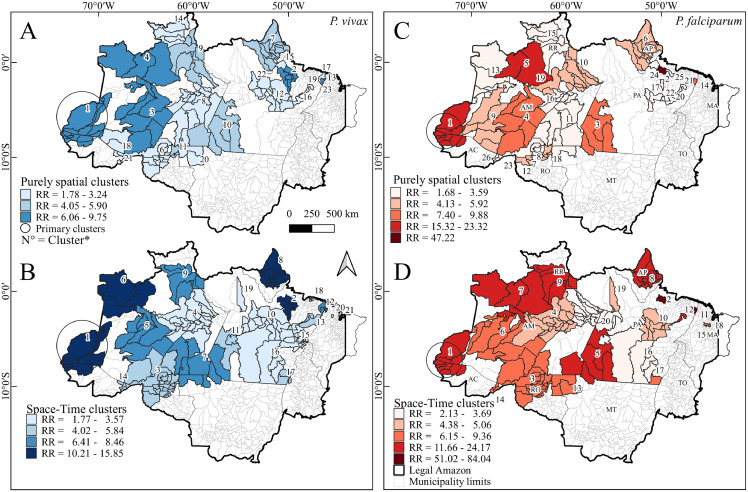
Spatial (above) and spatiotemporal (below) *P. vivax* (left) and *P. falciparum* (right) malaria relative risk (RR) clusters in the Brazilian Legal Amazon (2004-2018). The darkest cluster colours represent the clusters with the highest RR. Primary clusters are identified by a circle. The number of each cluster is detailed in Table S5 (*Appendix,* pp 12-13).

Throughout the analysed period (2004-2018), many municipalities had a consistently high incidence. In the study area, *P. vivax* and *P. falciparum* malaria were not distributed homogeneously. Several high-incidence spatial clusters were detected during the study period. São Gabriel da Cachoeira, Japurá, Atalaia do Norte and Barcelos municipalities (located in the Amazonas administrative zone) were the primary clusters (p<0·001). Secondary clusters were detected in Pará, Roraima, Acre, Rondônia and Amapá States (p<0·001) (Figure 3 and *Appendix*, pp 12-13).

The clusters with the highest relative risk (RR) found were: for *P. vivax*, for spatiotemporal analysis, in Pará state, in the municipalities of Anajás, Bagre, Breves, Curralinho, Limoeiro do Ajuru, Muaná, Oeiras do Pará and São Sebastião da Boa Vista (RR 2=15·85, 2009-2012); and in Amazonas state, in the municipalities of São Gabriel da Cachoeira, Japurá, Santa Isabel do Rio Negro (RR 6=12·67, 2012-2018); and for spatial analysis, in Amazonas, in the municipalities of Barcelos, Fonte Boa, Maraã, São Gabriel da Cachoeira and Santa Isabel do Rio Negro (RR 4=7·82); and in Pará state, in the municipalities of Anajás, Bagre, Breves, Curralinho, Limoeiro do Ajuru, Muaná, Oeiras do Pará and São Sebastião da Boa Vista (RR 2=7·4); and for *P. falciparum*, for spatiotemporal analyses, in Cachoeira do Piriá (RR 11=51·02, 2004-2006) and Anajás (RR 2=84·04, 2005-20111), both in Pará state; for spatial analysis, in Anajás (RR 2=47·22), in the state of Pará and, Barcelos and Santa Isabel do Rio Negro (RR 5=15·33), both in the state of Amazonas (Figure 3 and *Appendix*, pp 12-13).

### Malaria cases were more prevalent in young pregnant women

**Figure 4:**
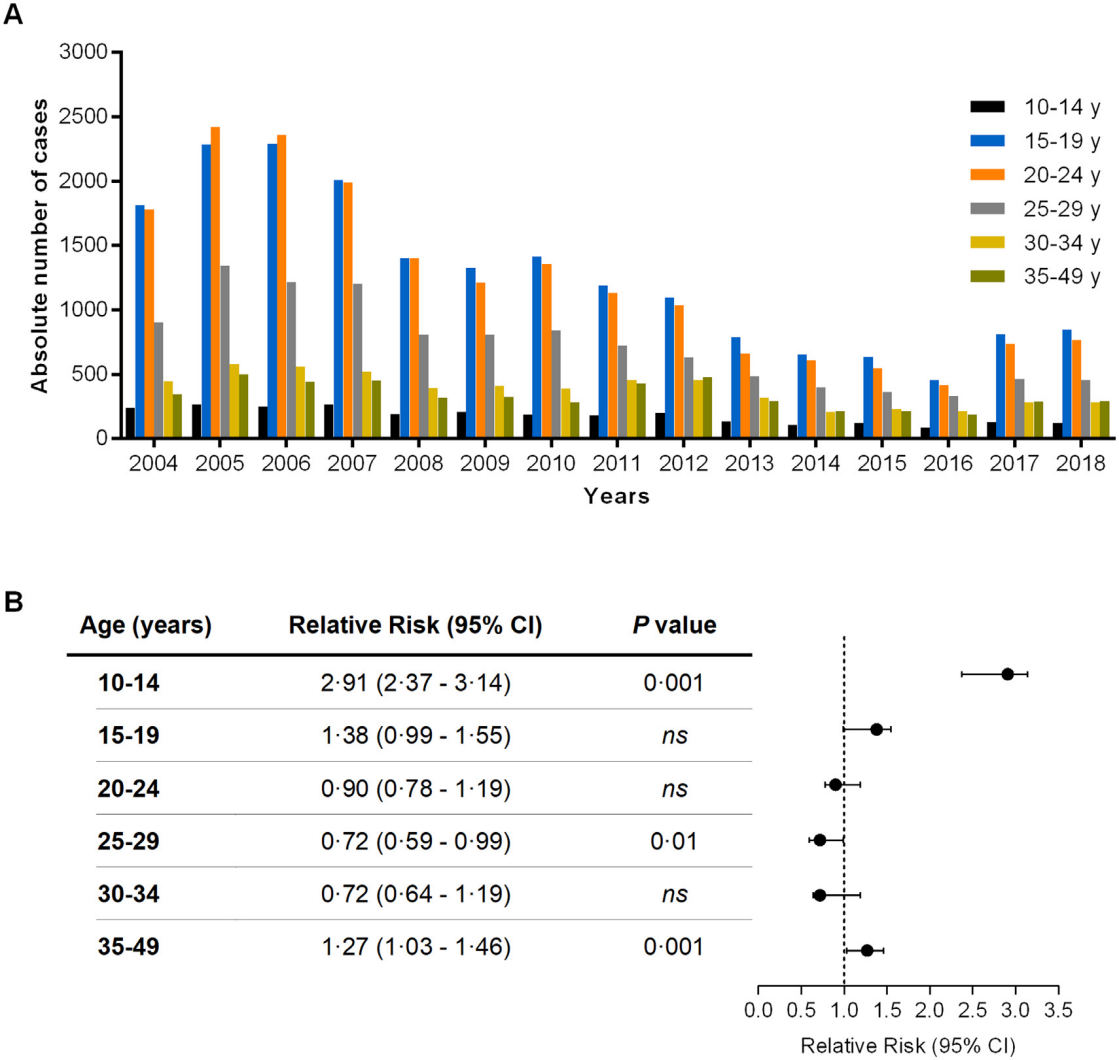
Distribution of malaria cases by maternal age, 2004-2018. (A) Time series of malaria cases in pregnant women aged 10-49 years old. (B) Relative risk of *Plasmodium* infection in pregnancy by age group. *ns*, nonsignificant. Number of pregnant women in each group: 10-14 years old, 2674; 15-19 years old, 18,998; 20-24 years old, 18,410; 25-29 years old, 10,967; 30-34 years old, 5740; and 35-49 years old, 5044.

Pregnant women were stratified according to age to assess which age group had the highest occurrence of infection (Figure 4). The age groups most affected by malaria consisted of younger women, which remained consistent over the years. Pregnant women aged 15–24 years old were the most affected, followed by women aged 25–29 years old (Figure 4A). Notably, during the 15-year study period, women aged 15 to 24 years old represented approximately 60·5% of the total population studied.

To understand whether this higher occurrence implied a higher risk of infection, we calculated the RR for each group (Figure 4B). We found that, although the highest concentration of malaria cases was among pregnant women aged 15 to 24 years old, the group at highest risk for infection was younger pregnant women aged 10 to 14 years old (Relative risk [RR]=2·91, 95% confidence interval 2·37 to 3·14, p=0·001) (Figure 4B).

### Treatment

Since only a small percentage of the evaluated notifications noted the prescription of appropriate medication for the pregnant woman (Table 1), we decided to evaluate this variable in depth. Nineteen treatment options are available according to the guidelines of the Brazilian Ministry of Health^15^^,^^16^ (*Appendix*, p 14), and the main options are summarized in Table 3. It was not possible to determine whether primaquine was being prescribed as a treatment in pregnant women specifically, as was observed in most schemes, or whether this number reflected notification filling errors. However, over the last three years, there has been substantial improvement in providing appropriate treatment for pregnant women (Table 3). It is possible to observe this improvement of approximately 280% when comparing the data from 2004 to 2015 with the values from 2016 to 2018.

**Table 3:** Description of the treatments reported during the entire analysis period and in the last three years.

	All years analysed (2004-18)	Last three years (2016-18)
	N=56870	N=7066
*Pv* infection, with chloroquine for 3 days and primaquine for 7 days	31064 (54·6)	2869 (40·6)
*Pm* infection in all ages and *Pv* infection in pregnant women and children under 6 months, with chloroquine for 3 days	6787 (11·9)	2451 (34·7)
*Pf* infection, with the combination for artemether + lumefantrine in 3 days	1277 (2·3)	369 (5·2)
*Pf* infection, with quinine for 3 days, doxycycline for 5 days and primaquine on the 6^th^ day	1578 (2·8)	6 (0·1)
*Pf* infection, with mefloquine as a single dose and primaquine on the second day	1233 (2·2)	-
*Pf* infection, with quinine for 7 days	1893 (3·3)	-
Other scheme used (by medical prescription)	10811 (19·0)	844 (11·9)
Others	2227 (3·9)	527 (7·5)

Abbreviations: N, total number of individuals; Pv, *Plasmodium vivax; Pf, Plasmodium falciparum*. The results are presented as the total number of events (n) and percentage (%).

### Malaria during pregnancy in the extra-Amazon region

In general, malaria cases outside the Amazon region account for approximately 1% of cases and are reported through the SINAN. Specifically, 13 cases of MiP were reported from 2007 to 2018, corresponding to 0·03% of notifications among pregnant women during this period. A map with the distribution of cases can be found in the *Appendix*, p 15. In contrast with the small number of cases in the population of pregnant women who live within the Amazon region, 50% of cases in the extra-Amazon region occurred in pregnant women older than 25 years of age.

Regarding species, 92·3% of the cases were caused by *P. vivax,* and only one case was caused by *P. falciparum*. Regarding treatment, only eight women had their treatment based on the guidelines of the Ministry of Health; four women (30·8%) underwent treatment with primaquine, and one woman received a scheme prescribed by a doctor (it is not possible to know which drug was used).

## Discussion

In a malaria-endemic country such as Brazil, having compulsory disease notification systems is essential for public health services to assess the scope of the problem and implement disease control measures. In addition, the implementation of these systems, in which the free distribution of antimalarials is associated with the registration of the case, opens the possibility of their use for descriptive epidemiological analyses, which can contribute to the improvement of health surveillance systems. In this work, we consolidated data on MiP in Brazil, with an emphasis on women living in the Amazon region, where approximately 99·9% of cases occur.

It is known that MiP imposes a significant economic burden, even in low-risk areas in Brazil.^17^ Costs associated with *P. vivax* infection are higher than costs associated with *P. falciparum* infection,^18^ which can cause multiple recurrences and is quite common in pregnant women. As expected and in accordance with other studies,^9^^,^^19^ our results showed that the highest incidence of malaria was caused by *P. vivax*. However, it is not possible to determine whether the cases were primary infections or recurrences since pregnant women cannot receive treatment with primaquine, and the diagnostic system used is not able to distinguish the status of infection.

Although there has been a decrease in the number of cases of MiP throughout the years, malaria eradication has been challenging. Pregnant women are reservoirs of *Plasmodium* spp. and can infect mosquito vectors, maintaining parasite transmission.^9^^,^^20^ Furthermore, the reduction in the number of recorded MiP cases does not make the issue less concerning. MiP in low-transmission areas can result in an increased risk of clinical disease with pronounced symptoms and malaria-related complications compared to MiP in high-transmission areas, where many pregnant women have acquired immunity.^3^ Associations between MiP and prematurity and small-for-gestational age have already been detected in low-transmission areas.^21^ Notably, the reduction in the number of cases from 2010 could be due to the free distribution of insecticide-treated nets (ITNs) to the municipalities with the heaviest malaria burdens, with the objective of reducing the incidence of the disease by up to 80% in the entire population, including pregnant women.^8^

In areas with low malaria transmission, parity has less of an impact on the risk of infection than in the high-transmission area; nevertheless, the risk is still greater in primigravidity.^22^^,^^23^ Therefore, in these areas, women lacking immunity, regardless of whether they are primigravid, are susceptible to malaria and have a higher risk of severe malaria and death when infected by *P. falciparum*.^3^ In some of the hotspots, this species accounted for 30% of the infections (e.g., Acre State). Recent studies in this Amazon region hotspot showed that both *P. falciparum* and *P. vivax* infections were associated with increased risks of several adverse gestational effects, such as prematurity, small head circumference, low birth weight, and reduced maternal haemoglobin.^24^, ^25^, ^26^

The analysis revealed several spatial and spatiotemporal clusters with significantly high malaria incidence rates in pregnant women. In general, we observed strong similarities between the hotspot municipalities in both the purely spatial and spatiotemporal malaria incidence clusters detected by the respective scan analysis. Undoubtedly, the statistically significant primary and secondary clusters identified in the spatial and spatiotemporal scan analyses corresponded with the areas of high malaria incidence risk identified by the smoothed SEB risk distribution maps. For instance, the smoothed risk distribution map identified a high concentration of malaria cases in Amazonas State, overlapping with the statistically significant spatial and spatiotemporal incidence clusters detected by the scan analyses. The result of the spatial analysis suggests a high malaria burden in these municipalities. It is important that the Health Surveillance Secretariat monitor whether the recommended actions and policies to combat malaria are being correctly applied in these areas. This process requires the urgent attention of local health managers for the prevention and control of malaria cases in the affected areas, reinforcing early diagnosis and timely treatment.

We observed two incidence patterns: annual and seasonal. However, when we compared the clusters, we found variations between them. Thus, local managers of malaria control programmes should be prepared to initiate interventions according to the epidemiological situation of the area. The decreases in the numbers of malaria cases after epidemics could occur because of the intensification of control measures, which has been reported in other areas.^27^

The spatial distribution that we observed in our analyses, especially the clusters formed in municipalities in the States of Amazonas and Pará (São Gabriel da Cachoeira and Oeiras do Pará, respectively), corroborates the results observed by other authors for the general population.^28^ This outcome shows that, despite the peculiarities of malaria during pregnancy, in regions at high risk for transmission, pregnant women are included in the same epidemiological context.

The stratification of cases by maternal age revealed that MiP in the endemic region was concentrated in younger women (15 to 24 years old), with the highest risk in those aged 10 to 14 years old, in accordance with other studies performed in Brazil.^29^, ^30^, ^31^ Lower maternal age is an additional risk factor for malaria in pregnancy since it is directly associated with primigravidity.^22^ Furthermore, maternal age has been identified as a risk factor in several studies, mainly due to increased susceptibility to the disease and increased risk of adverse pregnancy outcomes.^17^^,^^32^^,^^33^ However, outside the Amazon region, the most prevalent age group was older than 25 years old. An explanation could be that, in poorer or less developed regions, such as the Amazon region, the adolescent pregnancy rate is extremely high, increasing the risk of malaria and its adverse effects.^31^ It was not possible to determine the parity of women in the SIVEP-Malaria, rendering age–parity analysis impossible.

In Brazil, diagnosis and treatment with antimalarials are free. Briefly, the standard treatment for pregnant women with *P. vivax* malaria is chloroquine, and for *P. falciparum* infections, a combination of artemether and lumefantrine is used. Primaquine crosses the placenta and can cause haemolysis in a G6PD-deficient foetus; it is therefore not recommended for use during pregnancy or during breastfeeding unless the G6PD status of the infant is known. The prevalence of G6PD deficiency is approximately 4·5% in the Brazilian population, and the risk of haemolysis can be high when using primaquine to treat malaria.^34^^,^^35^ Finding new antimalarial drugs for use in pregnant women is one of the greatest challenges to fighting malaria in this population because of concerns about teratogenicity and embryotoxicity.^3^ Here, we note that, between 2016 and 2018, more than 40% of the treatments reported for pregnant women in Brazil included primaquine. It is important to emphasize that the Ministry of Health in Brazil does not recommend the use of primaquine for pregnant women and children younger than 6 months of age.^36^ Therefore, we believe that most of the results that we found regarding the use of primaquine during pregnancy could be errors in completing the forms or typing errors during information transfer to the system.

Additionally, regarding the treatment registered on the notification forms, other points should be considered. First, it was not possible to confirm whether primaquine was administered to pregnant women since, as pregnant women are considered a high-risk group, the drug could have been prescribed by the doctor; furthermore, there could have been a failure in communication between the doctor and the professional who completed the notification form. Second, it is possible that the drug was incorrectly dispensed by health professionals who did not comply with the country's malaria treatment guidelines for pregnant women, not only in the first trimester (when there is uncertainty about pregnancy status) but also in other gestational trimesters, an observation also reported in another study.^24^ Finally, as mentioned above, it is possible that there were form-filling and/or typing errors. Thus, these results and conclusions should be interpreted with great caution. This study analysed data registered in the Ministry of Health's information system, but it is important to understand its nuances and limitations.

Malaria cases in the extra-Amazon region account for only approximately 1% of reported cases in the total population, but there is great concern due to the higher mortality rate in these cases. In addition, studies of MiP in those regions are scarce.^37^ Here, we present an overview of MiP in women living outside the Amazon region. Information about these cases is extremely important for malaria control since diagnosis and treatment generally do not occur early, increasing the risk for both the mother and the foetus. Our data analysis also showed a large number of cases in which inappropriate treatment (treatment including primaquine) was reported, demonstrating a failure in the training of health professionals for accurate notification and the prescription of appropriate drugs for pregnant women.

The present study provides a national-scale overview of the MiP situation in Brazil based on robust data from the SIVEP-Malaria over a period of 15 years. This study is the first to provide insight into the spatial and temporal trends of the relative burden of autochthonous MiP. The use of geographic information systems and spatiotemporal geostatistics has become increasingly important in the guidance of malaria control programmes. This analysis could contribute to a better understanding of the dynamics of MiP in the country.

Some methodological limitations of this study must be noted. The data were obtained from a secondary source, in which there might have been modifications caused by the incorrect completion of notification forms, as well as typos when entering data into the online system (SIVEP-Malaria). This assumption is reinforced by the large numbers of ignored and incomplete fields and failure/errors in completing the forms. Regarding this point, the incompleteness or failure to complete the "pregnancy status" field deserves attention since our results could have underestimated the cases of MiP in the country, making the problem even more serious than what we presented in our study.

MiP causes serious problems for maternal-foetal dyads in the Brazilian Amazon region. In summary, despite the general reduction in the number of cases over the years, hotspots in Acre, Amazonas and Pará require attention from the Ministry of Health's malaria control programme. Reducing the number of cases in this population urgently requires the development of better screening and prevention strategies, as well as the development of drugs for complete treatment in pregnant women, especially those with *P. vivax* infections. In general, it is necessary to constantly train health teams to correctly complete notification forms and enter data into the system since the SIVEP-Malaria is extremely important for guiding public health policies.

## Contributors

JGD and CRFM designed the study. JGD, LCG, PM, SE and CRFM were involved in the data acquisition and scientific input. JGD, LCG, CL, RGSP, PM, SE and CRFM contributed to the analysis and/or interpretation of the data. JGD and LCG wrote the manuscript. CRFM was the main funder of this work, had full access to all of the data in the study and takes responsibility for the integrity of the data and the accuracy of the data analysis. All of the authors reviewed and approved the final version of this manuscript.

## Data sharing statement

Anonymous data will be made available to researchers who submit a request on the Brazilian Government Information Access Platform website: https://esic.cgu.gov.br/sistema/site/index.aspx. Additional data and other websites for accessing the other databases are available online as ***Supplementary Material***.

## Funding

This work was primarily funded by grants from the São Paulo Research Foundation-FAPESP (grants nos. 2018/20468-0 and 2020/06747-4 to CRFM, and 2017/05782-8 and 2020/03163-1 to SE) (www.fapesp.br) and The National Council for Scientific and Technological Development-CNPq (grants nos. 302917/2019-5 and 408636/2018-1 to CRFM, 304033/2021-9 to SE, and 409216/2018-6 to JGD) (www.cnpq.br). None of the granting agencies played any role in the design of the study; collection, analysis, or interpretation of data; or writing of the manuscript.

## Declaration of interests

The authors declare that they have no conflicts of interest.

## Appendix Supplementary materials

Image, application 1

Image, application 2
