# Supplementary material for: Mapping and characterizing areas with high levels of malaria in pregnancy in Brazil: A spatiotemporal analysis
Source: Lancet Reg Health Am. 2022 May 27;12:100285. doi: 10.1016/j.lana.2022.100285 (PMC9903888; doi:10.1016/j.lana.2022.100285)
Supplement: Supplementary file 1 [file mmc1.docx]

**Supplementary appendix**

**Mapping and characterizing areas with high levels of malaria in pregnancy in Brazil: A spatiotemporal analysis**

Jamille Gregório Dombrowski, PhD^a^, Laura Cordeiro Gomes, MSc^a^, Camila Lorenz, PhD^b^, Raquel Gardini Sanches Palasio, PhD^b^, Paola Marchesini, PhD^c^, Sabrina Epiphanio, PhD^d^, Claudio Romero Farias Marinho, PhD^a^

^a^ Department of Parasitology, Institute of Biomedical Sciences, University of São Paulo, São Paulo, Brazil

^b^ Department of Epidemiology, School of Public Health, University of São Paulo, São Paulo, Brazil

^c^ Department of Transmissible Diseases Surveillance, Ministry of Health, Brasília, Brazil.

^d^ Department of Clinical and Toxicological Analyses, School of Pharmaceutical Sciences, University of São Paulo, São Paulo, Brazil

**Additional description of the main study area (Brazilian Legal Amazon region).**

The Brazilian Legal Amazon region is located in South America and comprises 9 states: Acre, Amazonas, Rondônia, Roraima, Amapá, Pará, Mato Grosso, Tocantins and part of Maranhão (S1 Fig). This region has high endemicity for malaria, accounting for approximately 99% of all notified cases of the disease.^1^ Thus, only municipalities in the Legal Amazon report malaria cases through the Malaria Epidemiological Surveillance System (SIVEP-Malaria), which is a priority of the National Program for the Prevention and Control of Malaria of the Brazilian Ministry of Health.

**Figure S1. Map showing the Brazilian Legal Amazon region.**


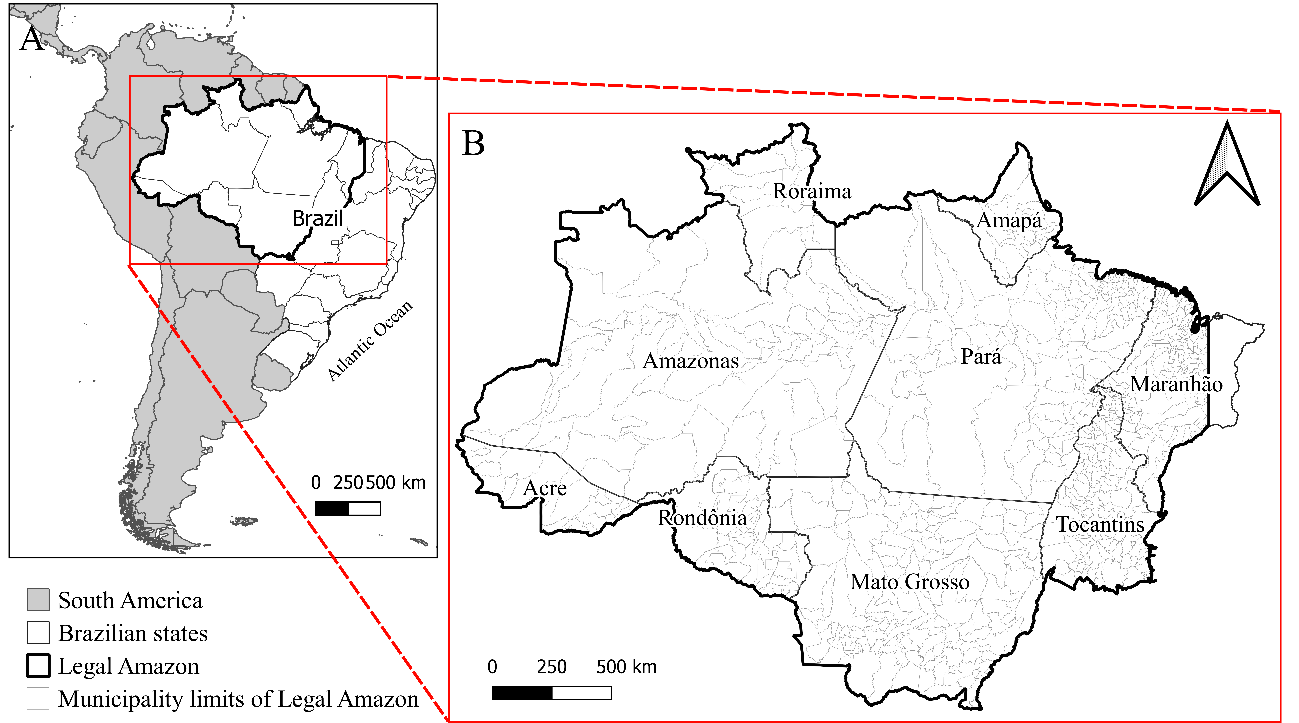


1 Ferreira MU, Castro MC. Challenges for malaria elimination in Brazil. *Malar J* 2016; **15**: 284.

**Table S1. Difference between municipality of residence and probable municipality of infection by year.**

| **Year** | **% of difference**  **Residence *vs.* Infection** | **Absolute numbers of cases** |
| --- | --- | --- |
| 2004 | 14·9 | 820 |
| 2005 | 13·1 | 968 |
| 2006 | 13·7 | 972 |
| 2007 | 11·1 | 717 |
| 2008 | 12·7 | 571 |
| 2009 | 11·0 | 470 |
| 2010 | 9·7 | 435 |
| 2011 | 8·8 | 360 |
| 2012 | 9·3 | 363 |
| 2013 | 9·1 | 243 |
| 2014 | 8·0 | 176 |
| 2015 | 6·8 | 144 |
| 2016 | 8·5 | 143 |
| 2017 | 9·1 | 248 |
| 2018 | 8·9 | 246 |
| **Mean** | **10·3** | **458·4** |

**Table S2. GATHER checklist of information that should be included in new reports of global health estimates.**

| Item # | Checklist item | Reported on page # |
| --- | --- | --- |
| Objectives and funding | | |
| 1 | Define the indicator(s), populations (including age, sex, and geographic entities), and time period(s) for which estimates were made. | 5 |
| 2 | List the funding sources for the work. | 3 |
| Data inputs | | |
| *For all data inputs from multiple sources that are synthesized as part of the study:* | | |
| 3 | Describe how the data were identified and how the data were accessed. | 5-6 |
| 4 | Specify the inclusion and exclusion criteria. Identify all ad hoc exclusions. | 5-7 |
| 5 | Provide information on all included data sources and their main characteristics. For each data source used, report reference information or contact name/institution, population represented, data collection method, year(s) of data collection, sex and age range, diagnostic criteria or measurement method, and sample size, as relevant. | 5-6 and  Supplementary pages 5-7 |
| 6 | Identify and describe any categories of input data that have potentially important biases (e.g., based on characteristics listed in item 5). | Supplementary pages 5-7 |
| *For data inputs that contribute to the analysis but were not synthesized as part of the study:* | | |
| 7 | Describe and provide sources for any other data inputs. | 6 and  Supplementary page 5 |
| *For all data inputs:* | | |
| 8 | Provide all data inputs in a file format from which data can be efficiently extracted (e.g., a spreadsheet rather than a PDF), including all relevant meta-data listed in item 5. For any data inputs that cannot be shared because of ethical or legal reasons, such as third-party ownership, provide a contact name or the name of the institution that retains the right to the data. | 6 and Supplementary pages 5, 14 |
| Data analysis | | |
| 9 | Provide a conceptual overview of the data analysis method. A diagram might be helpful. | 7-8 |
| 10 | Provide a detailed description of all steps of the analysis, including mathematical formulae. This description should cover, as relevant, data cleaning, data preprocessing, data adjustments and weighting of data sources, and mathematical or statistical model(s). | 7-8  Supplementary pages 6-7 and 12-13 |
| 11 | Describe how candidate models were evaluated and how the final model(s) was/were selected. | 7-8 |
| 12 | Provide the results of an evaluation of model performance, if performed, as well as the results of any relevant sensitivity analysis. | Supplementary pages 12-13 |
| 13 | Describe methods for calculating uncertainty of the estimates. State which sources of uncertainty were, and were not, accounted for in the uncertainty analysis. | 7-8 |
| 14 | State how the analytic or statistical source code used to generate estimates can be accessed. | Supplementary page 8, 12-13 |
| Results and discussion | | |
| 15 | Provide published estimates in a file format from which data can be efficiently extracted. | - |
| 16 | Report a quantitative measure of the uncertainty of the estimates (e.g., uncertainty intervals). | 8-13  Figures 1-4 |
| 17 | Interpret results in light of existing evidence. If updating a previous set of estimates, describe the reasons for changes in estimates. | 13-18 |
| 18 | Discuss limitations of the estimates. Include a discussion of any modelling assumptions or data limitations that affect interpretation of the estimates. | 17 |

**Access to databases (SINASC, SIM and IBGE)**

For this manuscript, three other information systems of the Brazilian Ministry of Health were used

1) Birth Information System (SINASC) - <http://tabnet.datasus.gov.br/cgi/deftohtm.exe?sinasc/cnv/nvbr.def>

2) Mortality Information System (SIM) - <http://tabnet.datasus.gov.br/cgi/tabcgi.exe?sim/cnv/fet10uf.def>

3) Brazilian Institute of Geography and Statistics (IBGE) - <http://tabnet.datasus.gov.br/cgi/deftohtm.exe?ibge/cnv/projpopuf.def>

Data from the SINASC and SIM were used to calculate the estimated number of pregnant women per municipality. For the calculation, we totalled the total number of live births and the number of foetal deaths by municipality of maternal residence and year. To estimate the female population by municipality and year, information from the IBGE was used. For the three information systems, the age range was 10 to 49 years old.

**Variables of the Epidemiological Surveillance Information System - Malaria (SIVEP-Malaria)**

The malaria notification form has approximately 40 variables, but only the 15 variables described in Table S3 were analysed in this study. In addition, these variables were common throughout all of the years studied (2004-2018) and were thus used to standardize the datasets.

**Table S3. Variables available for analysis in the malaria notification form present in all years.**

| **Variables** | **Description** |
| --- | --- |
| DT_NOTIF | Notification date |
| SEM_NOTI | Notification epidemiological week |
| TIPO_LAM | Detection type (passive or active) |
| UF_NOTIF | Patient notification UF according to the IBGE code |
| MUN_NOTI | Code of the notifying municipality according to the IBGE code |
| ID_PACIE | Patient age |
| NIV_ESCO | Patient education level |
| UF_RESID | UF of residence of the patient according to the IBGE code |
| MUN_RESI | Municipality code referring to the patient's address according to IBGE |
| SINTOMAS | Presence of symptoms |
| COD_OCUP | Main occupation performed by the patient over the last 15 days |
| UF_INFEC | Probable UF of infection according to the IBGE code |
| MUN_INFE | Probable municipality of infection according to the IBGE code |
| RES_EXAM | Result of the exam after analysis of the thick drop test |
| ESQUEMA | Treatment scheme used |

**Cleaning of the SIVEP-Malaria database**

Step by step of the database clean-up process:

1^st^ - Filter by the SEX variable and exclude all males (M).

| **Year** | **Total initial number (N) of records** | **Final N without M** |
| --- | --- | --- |
| 2004 | 560,431 | 195,175 |
| 2005 | 757,514 | 277,307 |
| 2006 | 695,190 | 261,404 |
| 2007 | 558,598 | 212,717 (8 empty) |
| 2008 | 376,977 | 144,883 (2 empty) |
| 2009 | 370,558 | 141,722 |
| 2010 | 414,849 | 159,881 (1 empty) |
| 2011 | 317,207 | 121,876 |
| 2012 | 280,995 | 107,942 |
| 2013 | 205,432 | 79,536 |
| 2014 | 163,697 | 64,656 (2 empty) |
| 2015 | 165,828 | 67,119 |
| 2016 | 151,622 | 60,541 |
| 2017 | 237,955 | 95,250 |
| 2018 | 243,182 | 96,182 |

2^nd^ - Selection of women of childbearing age: exclusion - Age < 10 years old and then > 49 years old and age-related typing errors.

| **Year** | **Total initial number (N) of records** | **Final N**  **< 10 years old** | **Final N**  **> 49 years old** |
| --- | --- | --- | --- |
| 2004 | 195,175 | 140,898 | 126,495 - Sex I* = 126,466 |
| 2005 | 277,307 | 197,890 | 176,988 - Sex I* = 176,913 |
| 2006 | 261,404 | 184,441 | 165,560 - Sex I* = 165,484 |
| 2007 | 212,717 | 150,283 | 134,881 - Sex I* = 134,843 |
| 2008 | 144,883 | 100,323 | 89,791 - Sex I* = 89,738 |
| 2009 | 141,722 | 97,936 | 87,736 - Sex I* = 87,693 |
| 2010 | 159,881 | 112,587 | 100,972 - Sex I* = 100,934 |
| 2011 | 121,876 | 87,447 | 77,808 - Sex I* = 77,788 |
| 2012 | 107,942 | 77,315 | 68,393 - Sex I* = 68,391 |
| 2013 | 79,536 | 59,355 | 52,381 |
| 2014 | 64,656 | 48,172 | 42,335 |
| 2015 | 67,119 | 49,017 | 42,919 |
| 2016 | 60,541 | 44,745 | 38,811 - Sex I* = 38,809 |
| 2017 | 95,250 | 71,189 | 61,378 |
| 2018 | 96,182 | 71,705 | 61,977 |

*Exclusion of people with undetermined sex (I), leaving only those with the variable PREGNANT1=1 and/or PREGNANT from 1 to 4.

3^rd^ - Exclusion of LVC (through the TIPO_LAM column) until 2010. In 2011, there was a change in the form, and the exclusion also uses the ID_LVC column.

| **Year** | **Total initial number (N) of records** | **Final N**  **without LVC** |
| --- | --- | --- |
| 2004 | 126,466 | 105,394 |
| 2005 | 176,913 | 142,106 |
| 2006 | 165,484 | 130,915 |
| 2007 | 134,843 | 110,388 |
| 2008 | 89,738 | 75,129 |
| 2009 | 87,693 | 73,537 |
| 2010 | 100,934 | 81,582 |
| 2011 | 77,788 | 65,804 |
| 2012 | 68,391 | 59,210 |
| 2013 | 52,381 | 45,606 |
| 2014 | 42,335 | 37,367 |
| 2015 | 42,919 | 37,139 |
| 2016 | 38,809 | 33,161 |
| 2017 | 61,378 | 50,702 |
| 2018 | 61,977 | 49,977 |

**Note:**

For the analyses, all women were entered with the variable PREGNANT1 filled in with the number 1 and the variable PREGNANT filled in with the numbers 1 to 4. From 2011 onwards, there was a change in the notification form, so from that year, the variable PREGNANT was considered the main variable. Thus, women who were filled in with the numbers 5 or 6 were excluded, even if the variable PREGNANT1 was filled in with the number 1, which was considered a typing error.

4th - Two selections were also made before the analyses:

1) Selection of pregnant women residing in Brazil (variable: PAIS_RES);

2) Selection of pregnant women where the country of infection was Brazil (variable: PAIS_INF).

| **Year** | **Total initial number (N) of records** | **Total N**  **Pregnant woman** | **Total N**  **Pregnant woman (Brazil)** |
| --- | --- | --- | --- |
| 2004 | 105,394 | 5674 | 5520 |
| 2005 | 142,106 | 7577 | 7393 |
| 2006 | 130,915 | 7332 | 7111 |
| 2007 | 110,388 | 6657 | 6433 |
| 2008 | 75,129 | 4663 | 4503 |
| 2009 | 73,537 | 4400 | 4278 |
| 2010 | 81,582 | 4624 | 4467 |
| 2011 | 65,804 | 4182 | 4108 |
| 2012 | 59,210 | 4021 | 3895 |
| 2013 | 45,606 | 2856 | 2681 |
| 2014 | 37,367 | 2286 | 2189 |
| 2015 | 37,139 | 2233 | 2104 |
| 2016 | 33,161 | 1802 | 1681 |
| 2017 | 50,702 | 2812 | 2714 |
| 2018 | 49,977 | 2884 | 2756 |

**Table S4. Annual distribution of malaria cases among women aged 10-49 years old during the study period according to data from the SIVEP-Malaria.**

| **Year** |  | **Total malaria** | | | | ***P. vivax*** | | | | ***P. falciparum*** | | | |
| --- | --- | --- | --- | --- | --- | --- | --- | --- | --- | --- | --- | --- | --- |
|  | **Total malaria**  **in women** | **Nonpregnant** | | **Pregnant** | | **Nonpregnant** | | **Pregnant** | | **Nonpregnant** | | **Pregnant** | |
|  | **N** | **N** | **%** | **N** | **%** | **N** | **%** | **N** | **%** | **N** | **%** | **N** | **%** |
| **2004** | 102,687 | 97,167 | 94·6 | 5520 | 5·4 | 74,193 | 76·4 | 4036 | 73·1 | 22,012 | 22·7 | 1398 | 25·3 |
| **2005** | 139,581 | 132,188 | 94·7 | 7393 | 5·3 | 98,227 | 74·3 | 5108 | 69·1 | 32,480 | 24·6 | 2173 | 29·4 |
| **2006** | 128,164 | 121,053 | 94·5 | 7111 | 5·5 | 88,238 | 72·9 | 4875 | 68·6 | 31,081 | 25·7 | 2098 | 29·5 |
| **2007** | 107,760 | 101,327 | 94·0 | 6433 | 6·0 | 80,554 | 79·5 | 4865 | 75·6 | 19,925 | 19·7 | 1488 | 23·1 |
| **2008** | 73,291 | 68,788 | 93·9 | 4503 | 6·1 | 58,100 | 84·5 | 3659 | 81·3 | 10,162 | 14·8 | 808 | 17·9 |
| **2009** | 71,688 | 67,410 | 94·0 | 4278 | 6·0 | 56,429 | 83·7 | 3410 | 79·7 | 10,435 | 15·5 | 821 | 19·2 |
| **2010** | 79,364 | 74,897 | 94·4 | 4467 | 5·6 | 63,594 | 84·9 | 3612 | 80·9 | 10,621 | 14·2 | 811 | 18·2 |
| **2011** | 64,346 | 60,238 | 93·6 | 4108 | 6·4 | 52,565 | 87·3 | 3460 | 84·2 | 6977 | 11·6 | 600 | 14·6 |
| **2012** | 57,337 | 53,442 | 93·2 | 3895 | 6·8 | 45,182 | 84·5 | 3170 | 81·4 | 6768 | 12·7 | 621 | 15·9 |
| **2013** | 43,310 | 40,629 | 93·8 | 2681 | 6·2 | 32,985 | 81·2 | 2113 | 78·8 | 6564 | 16·2 | 512 | 19·1 |
| **2014** | 36,092 | 33,903 | 93·9 | 2189 | 6·1 | 28,216 | 83·2 | 1718 | 78·5 | 5366 | 15·8 | 446 | 20·4 |
| **2015** | 35,777 | 33,673 | 94·1 | 2104 | 5·9 | 29,536 | 87·7 | 1750 | 83·2 | 3638 | 10·8 | 318 | 15·1 |
| **2016** | 31,810 | 30,129 | 94·7 | 1681 | 5·3 | 26,302 | 87·3 | 1436 | 85·4 | 3334 | 11·1 | 214 | 12·7 |
| **2017** | 49,531 | 46,817 | 94·5 | 2714 | 5·5 | 41,168 | 87·9 | 2318 | 85·4 | 4844 | 10·3 | 326 | 12·0 |
| **2018** | 48,189 | 45,433 | 94·3 | 2756 | 5·7 | 39,869 | 87·8 | 2337 | 84·8 | 4454 | 9·8 | 356 | 12·9 |
| **Total** | **1,068.927** | **1,007,094** | **94·2** | **61,833** | **5·8** | **815,158** | **80·9** | **47867** | **77·4** | **178,661** | **17·7** | **12,990** | **21·0** |

**Figure S2. Spatial distribution of *P. vivax* malaria in pregnancy, Brazilian Amazon Legal, 2004-2018.**


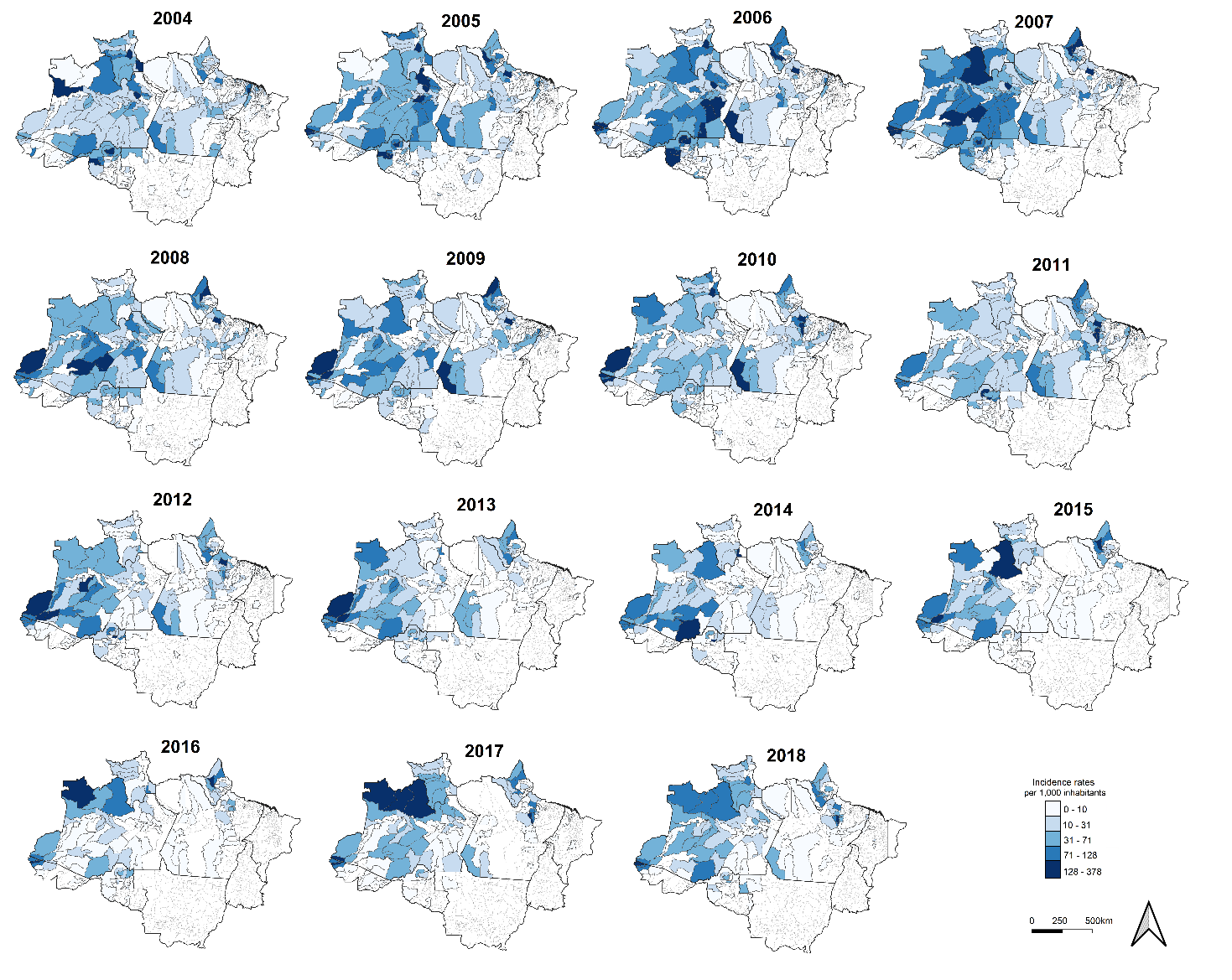


**Figure S3. Spatial distribution of *P. falciparum* malaria in pregnancy, Brazilian Amazon Legal, 2004-2018.**


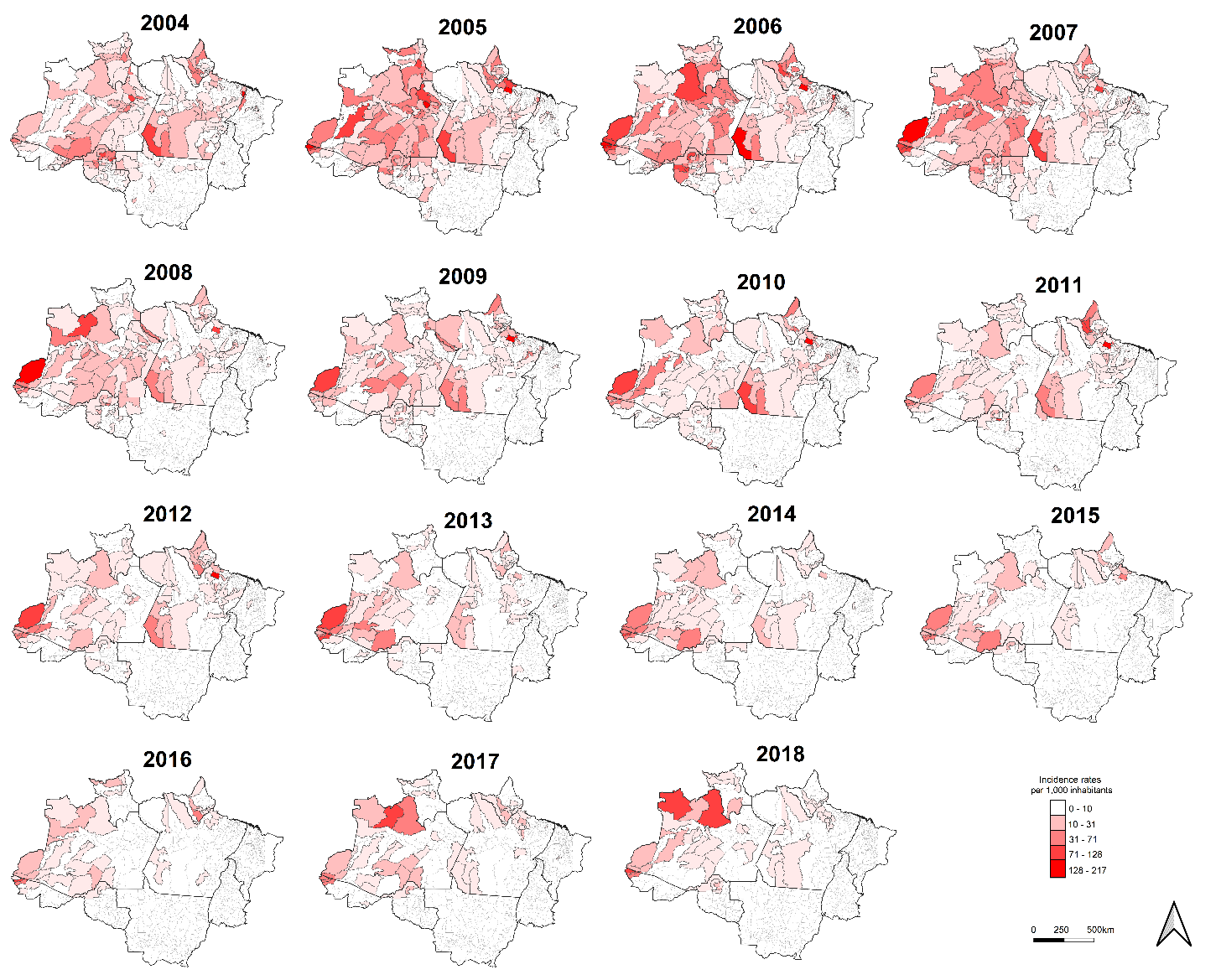


**Figure S4. Monthly distribution of total malaria cases in the populations of pregnant and nonpregnant women, 2016-2018.**





**Table S5. Spatiotemporal analysis of malaria in pregnancy clusters over the study period, 2004-2018.**

| *P. vivax* | | | | | | |
| --- | --- | --- | --- | --- | --- | --- |
| **Spatiotemporal analysis** | | | | | | |
| **Cluster** | **Start date** | **End date** | **Observed cases** | **Expected cases** | **Relative**  **Risk** | **p value** |
| 1 | 2007 | 2013 | 4505 | 454·57 | 10·84 | 0·001 |
| 2 | 2009 | 2012 | 2540 | 168·62 | 15·85 | 0·001 |
| 3 | 2004 | 2010 | 4291 | 794·14 | 5·84 | 0·001 |
| 4 | 2004 | 2008 | 5509 | 1681·65 | 3·57 | 0·001 |
| 5 | 2007 | 2013 | 1960 | 267·10 | 7·61 | 0·001 |
| 6 | 2012 | 2018 | 1008 | 79·56 | 12·92 | 0·001 |
| 7 | 2004 | 2010 | 1098 | 132·42 | 8·46 | 0·001 |
| 8 | 2005 | 2011 | 884 | 88·09 | 10·21 | 0·001 |
| 9 | 2004 | 2010 | 560 | 86·35 | 6·55 | 0·001 |
| 10 | 2005 | 2011 | 890 | 288·89 | 3·12 | 0·001 |
| 11 | 2006 | 2012 | 505 | 126·69 | 4·02 | 0·001 |
| 12 | 2004 | 2006 | 280 | 43·87 | 6·41 | 0·001 |
| 13 | 2008 | 2010 | 234 | 54·85 | 4·28 | 0·001 |
| 14 | 2004 | 2007 | 163 | 30·23 | 5·41 | 0·001 |
| 15 | 2004 | 2006 | 620 | 352·90 | 1·77 | 0·001 |
| 16 | 2004 | 2006 | 219 | 83·51 | 2·63 | 0·001 |
| 17 | 2004 | 2005 | 69 | 12·37 | 5·59 | 0·001 |
| 18 | 2005 | 2006 | 67 | 13·03 | 5·15 | 0·001 |
| 19 | 2004 | 2006 | 87 | 30·44 | 2·86 | 0·001 |
| 20 | 2007 | 2011 | 34 | 9·72 | 3·50 | 0·001 |
| 21 | 2004 | 2007 | 30 | 9·78 | 3·07 | 0·031 |
| **Spatial analysis** | | | | | | |
| **Cluster*** | **Observed cases** | **Expected cases** | **Relative**  **Risk** | **p value** |  |  |
| 1 | 8010·00 | 966·39 | 9·75 | 0·001 |  |  |
| 2 | 4484·00 | 658·75 | 7·41 | 0·001 |  |  |
| 3 | 3783·00 | 585·89 | 6·93 | 0·001 |  |  |
| 4 | 2157·00 | 286·98 | 7·82 | 0·001 |  |  |
| 5 | 4407·00 | 1448·90 | 3·25 | 0·001* |  |  |
| 6 | 4108·00 | 1347·87 | 3·24 | 0·001 |  |  |
| 7 | 1911·00 | 335·17 | 5·90 | 0·001 |  |  |
| 8 | 3450·00 | 1184·39 | 3·06 | 0·001 |  |  |
| 9 | 1324·00 | 268·12 | 5·05 | 0·001 |  |  |
| 10 | 1525·00 | 385·87 | 4·05 | 0·001 |  |  |
| 11 | 348·00 | 65·87 | 5·31 | 0·001 |  |  |
| 12 | 1479·00 | 756·60 | 1·99 | 0·001 |  |  |
| 13 | 194·00 | 32·01 | 6·08 | 0·001 |  |  |
| 14 | 441·00 | 164·18 | 2·70 | 0·001 |  |  |
| 15 | 629·00 | 312·18 | 2·03 | 0·001 |  |  |
| 16 | 220·00 | 71·55 | 3·08 | 0·001 |  |  |
| 17 | 314·00 | 148·59 | 2·12 | 0·001 |  |  |
| 18 | 315·00 | 156·67 | 2·02 | 0·001 |  |  |
| 19 | 153·00 | 58·82 | 2·61 | 0·001 |  |  |
| 20 | 184·00 | 87·91 | 2·10 | 0·001 |  |  |
| 21 | 129·00 | 55·69 | 2·32 | 0·001 |  |  |
| 22 | 245·00 | 137·66 | 1·78 | 0·001 |  |  |
| 23 | 76·00 | 40·99 | 1·86 | 0·006 |  |  |
|  |  |  |  |  |  |  |
| *P. falciparum* | | | | | | |
| **Spatiotemporal analysis** | | | | | | |
| **Cluster** | **Start date** | **End date** | **Observed cases** | **Expected cases** | **Relative**  **Risk** | **p value** |
| 1 | 2005 | 2011 | 1629 | 75·89 | 24·17 | 0·001 |
| 2 | 2005 | 2011 | 737 | 9·25 | 84·04 | 0·001 |
| 3 | 2004 | 2007 | 1131 | 130·20 | 9·36 | 0·001 |
| 4 | 2004 | 2007 | 1698 | 371·64 | 5·06 | 0·001 |
| 5 | 2004 | 2010 | 758 | 55·83 | 14·30 | 0·001 |
| 6 | 2005 | 2009 | 489 | 63·37 | 7·96 | 0·001 |
| 7 | 2017 | 2018 | 239 | 12·66 | 19·19 | 0·001 |
| 8 | 2005 | 2011 | 294 | 25·70 | 11·66 | 0·001 |
| 9 | 2004 | 2007 | 166 | 14·05 | 11·94 | 0·001 |
| 10 | 2004 | 2007 | 292 | 63·04 | 4·71 | 0·001 |
| 11 | 2004 | 2006 | 70 | 1·38 | 51·02 | 0·001 |
| 12 | 2008 | 2010 | 42 | 3·29 | 12·80 | 0·001 |
| 13 | 2005 | 2008 | 62 | 9·40 | 6·62 | 0·001 |
| 14 | 2004 | 2007 | 48 | 6·60 | 7·30 | 0·001 |
| 15 | 2005 | 2007 | 30 | 3·54 | 8·50 | 0·001 |
| 16 | 2004 | 2005 | 59 | 16·02 | 3·69 | 0·001 |
| 17 | 2004 | 2005 | 17 | 2·77 | 6·15 | 0·002 |
| 18 | 2004 | 2004 | 12 | 1·32 | 9·07 | 0·003 |
| 19 | 2010 | 2011 | 21 | 4·80 | 4·38 | 0·012 |
| 20 | 2005 | 2005 | 65 | 30·62 | 2·13 | 0·014 |
| **Spatial analysis** | | | | | | |
| **Cluster** | **Observed cases** | **Expected cases** | **Rel**  **Risk** | **p value** |  |  |
| 1 | 2936·00 | 157·62 | 23·32 | 0·001 |  |  |
| 2 | 939·00 | 21·29 | 47·22 | 0·001 |  |  |
| 3 | 978·00 | 105·57 | 9·89 | 0·001 |  |  |
| 4 | 1054·00 | 152·46 | 7·40 | 0·001 |  |  |
| 5 | 319·00 | 21·27 | 15·33 | 0·001 |  |  |
| 6 | 559·00 | 97·80 | 5·91 | 0·001 |  |  |
| 7 | 1136·00 | 422·76 | 2·84 | 0·001 |  |  |
| 8 | 805·00 | 274·84 | 3·05 | 0·001 |  |  |
| 9 | 445·00 | 94·75 | 4·82 | 0·001 |  |  |
| 10 | 341·00 | 77·69 | 4·47 | 0·001 |  |  |
| 11 | 423·00 | 120·55 | 3·59 | 0·001 |  |  |
| 12 | 190·00 | 36·63 | 5·24 | 0·001 |  |  |
| 13 | 353·00 | 137·91 | 2·60 | 0·001 |  |  |
| 14 | 87·00 | 9·34 | 9·37 | 0·001 |  |  |
| 15 | 205·00 | 58·46 | 3·54 | 0·001 |  |  |
| 16 | 318·00 | 143·53 | 2·24 | 0·001 |  |  |
| 17 | 242·00 | 102·01 | 2·40 | 0·001 |  |  |
| 18 | 95·00 | 23·14 | 4·13 | 0·001 |  |  |
| 19 | 59·00 | 14·16 | 4·18 | 0·001 |  |  |
| 20 | 64·00 | 20·88 | 3·07 | 0·001 |  |  |
| 21 | 54·00 | 17·16 | 3·15 | 0·001 |  |  |
| 22 | 60·00 | 22·62 | 2·66 | 0·001 |  |  |
| 23 | 69·00 | 29·11 | 2·38 | 0·001 |  |  |
| 24 | 152·00 | 91·09 | 1·68 | 0·001 |  |  |
| 25 | 32·00 | 9·37 | 3·42 | 0·001 |  |  |
| 26 | 74·00 | 38·77 | 1·91 | 0·002 |  |  |

*Cluster 5 obtained False Gini indexes, so it was disregarded, and it was superimposed on cluster 6.

**Table S6. Treatment schedule options available on the malaria notification form.**

| **N** | **Notification number - treatment schedule** |
| --- | --- |
| 1 | 1 - *Pv* or *Po* infection, with chloroquine for 3 days and primaquine for 7 days (short scheme) |
| 2 | 2 - *Pv* or *Po* infection, with chloroquine for 3 days and primaquine for 14 days (long scheme) |
| 3 | 3 - Infection by *Pm* in all ages and by *Pv* or *Po* in pregnant women and children younger than 6 months old, with chloroquine for 3 days |
| 4 | 4 - Prevention of frequent relapses by *Pv* or *Po*, with weekly chloroquine for 12 weeks |
| 5 | 5 - *Pf* infection, with the combination of artemether + lumefantrine for 3 days |
| 6 | 6 - *Pf* infection, with the combination of artesunate + mefloquine for 3 days |
| 7 | 7 - *Pf* infection, with quinine for 3 days, doxycycline for 5 days and primaquine on the 6th day |
| 8 | 8 - Mixed infection by *Pf* + *Pv* or *Po*, with artemether + lumefantrine or artesunate + mefloquine for 3 days and primaquine for 7 days |
| 9 | 9 - Uncomplicated *Pf* infections in the 1st trimester of pregnancy and in children younger than 6 months old, with quinine for 3 days and clindamycin for 5 days |
| 10 | 10 - Severe malaria complicated by *Pf* in all age groups |
| 11 | 11 - *Pf* infection, with the combination of artemether + lumefantrine for 3 days and primaquine as a single dose |
| 12 | 12 - *Pf* infection, with the combination of artesunate + mefloquine for 3 days and primaquine as a single dose |
| 13 | 83 - Mixed infection by *Pv* + *Pf*, with mefloquine as a single dose and primaquine for 7 days |
| 14 | 85 - *Pv* infection in children with vomiting, with artesunate rectal capsules for 4 days and primaquine for 7 days |
| 15 | 86 - *Pf* infection, with a single dose of mefloquine and primaquine on the second day |
| 16 | 87 - *Pf* infection, with quinine for 7 days |
| 17 | 88 - *Pf* infection in children, with artesunate rectal capsules for 4 days, a single dose of mefloquine on the 3rd day and primaquine on the 5th day |
| 18 | 89 - Mixed infection by *Pv* + *Pf*, with quinine for 3 days, doxycycline for 5 days and primaquine for 7 days |
| 19 | 99 - Another scheme (by physician) |

Abbreviations: Pv, *Plasmodium vivax*; Po, *Plasmodium ovale*; Pm*, Plasmodium malariae*; Pf, *Plasmodium falciparum*.

The correct treatment for malaria in pregnancy is described in the Ministry of Health guidelines. In short, for a noncomplicated *P. vivax* infection in pregnant women, it is recommended to use only chloroquine (150 mg) in a three-day treatment schedule for the acute phase (10 mg/kg on day 1 and 7.5 mg/kg on days 2 and 3) and weekly chloroquine (5 mg/kg/dose) until the end of pregnancy. For infections by *P. falciparum*, the use of artemether (20 mg) and lumefantrine (120 mg) in a three-day schedule, in a fixed combination, is recommended throughout pregnancy.

**Figure S5. Map with the distribution of autochthonous malaria in pregnancy in the extra-Amazon region, 2007-2018.**


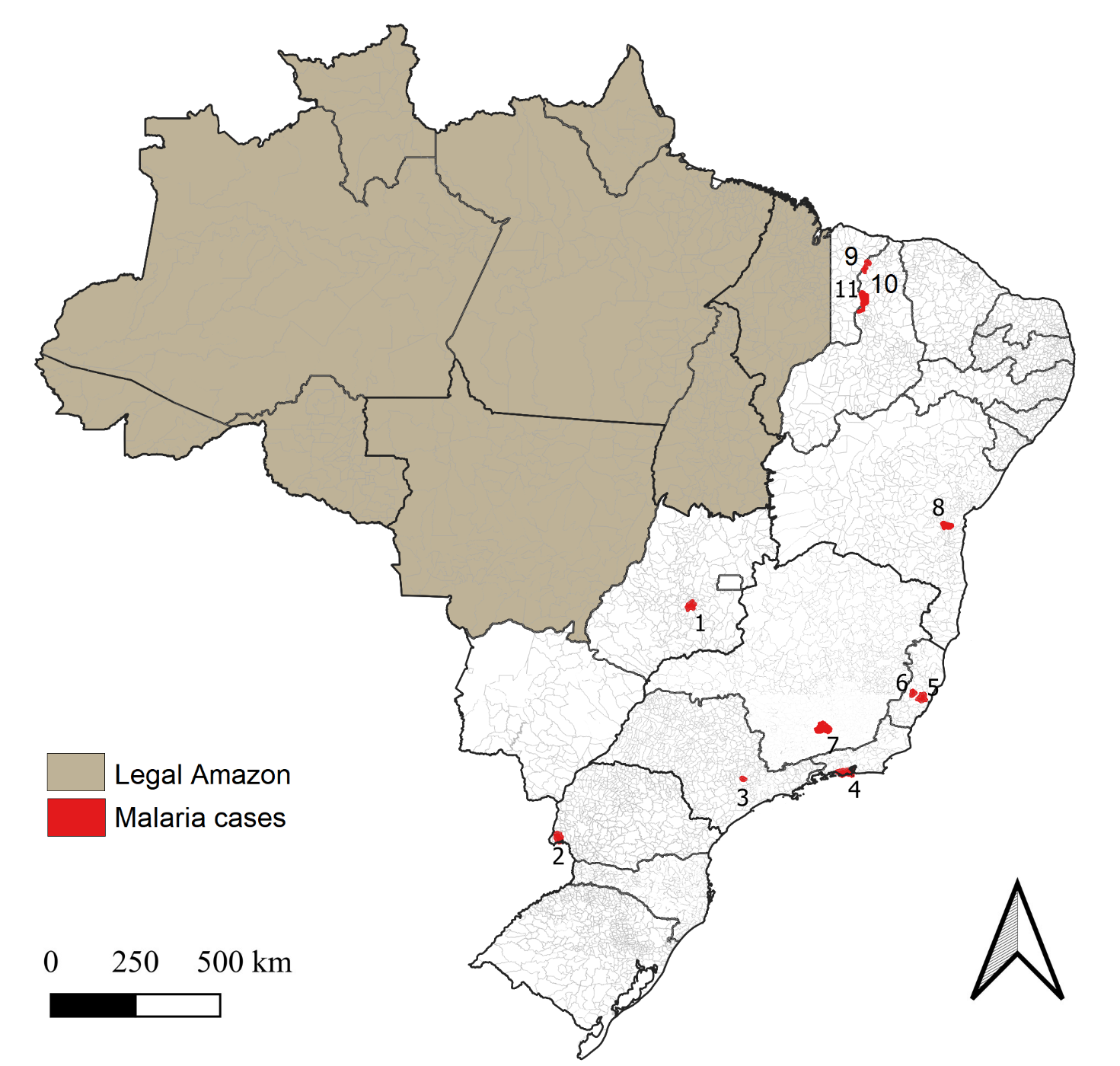


Municipalities with extra-Amazonian malaria cases between 2007 and 2018: (1) Goiânia/GO, (2) São Miguel do Iguaçu/PR, (3) Salto/SP, (4) Rio de Janeiro/RJ, (5) Santa Leopoldina/ES, (6) Itarana/ES, (7) São João Del Rei/MG, (8) Wenceslau Guimarães/BA, (9) Matias Olímpio/PI, (10) Teresina/PI, and (11) Porto/PI. Only São Miguel do Iguaçu/PR and Goiânia/GO reported 2 cases during the study period, and the others reported only 1 case.

Abbreviations of the names of the states: GO, Goiás; PR, Paraná; SP, São Paulo; RJ, Rio de Janeiro; ES, Espírito Santo; MG, Minas Gerais; BA, Bahia; PI, Piauí.
