# Supplementary material for: Mapping and characterizing areas with high levels of malaria in pregnancy in Brazil: A spatiotemporal analysis
Source: Lancet Reg Health Am. 2022 May 27;12:100285. doi: 10.1016/j.lana.2022.100285 (PMC9903888; doi:10.1016/j.lana.2022.100285)
Supplement: Supplementary file 2 [file mmc2.docx]

**Caption for supplementary material**

1. **Additional description of the main study area (Brazilian Legal Amazon region).**
2. **Figure S1.** Map showing the Brazilian Legal Amazon region.
3. **Table S1.** Difference between municipality of residence and probable municipality of infection by year.
4. **Table S2.** GATHER checklist of information that should be included in new reports of global health estimates.
5. **Access to databases (SINASC, SIM and IBGE)**
6. **Table S3.** Variables available for analysis in the malaria notification form present in all years.
7. **Cleaning of the SIVEP-Malaria database.**
8. **Table S4.** Annual distribution of malaria cases among women aged 10-49 years old during the study period according to data from the SIVEP-Malaria.
9. **Figure S2.** Spatial distribution of *P. vivax* malaria in pregnancy, Brazilian Amazon Legal, 2004-2018.
10. **Figure S3.** Spatial distribution of *P. falciparum* malaria in pregnancy, Brazilian Amazon Legal, 2004-2018.
11. **Figure S4.** Monthly distribution of total malaria cases in the populations of pregnant and nonpregnant women, 2016-2018.
12. **Table S5.** Spatiotemporal analysis of malaria in pregnancy clusters over the study period, 2004-2018.
13. **Table S6.** Treatment schedule options available on the malaria notification form.
14. **Figure S5.** Map with the distribution of autochthonous malaria in pregnancy in the extra-Amazon region, 2007-2018.
